# Topics of the Month

**Published:** 1893-09

**Authors:** 


### TOPICS OF THE MONTH.

Mr. Ernest Hart, the distinguished editor of the *British Medical Journal*, paid a visit to this country in the early Summer, and attended the annual meeting of the American Medical Association, at Milwaukee, in June. During the course of a very able address which Mr. Hart delivered at the Medical Editors' meeting on Monday evening, June 5, 1893, he took occasion to refer to consultations with homeopaths. Whatever may have been the practice in England with regard to this subject, and upon which Mr. Hart animadverted, there has never been in this country any considerable danger of such an admixture of methods that are as diametrically opposed as the poles.

Somebody must have given Mr. Hart an erroneous view of the ethical conditions surrounding the American medical profession. We infer that he has been "coached" with reference to the attitude of the American Medical Association towards the Medical Society of the State of New York.

Whoever has gratuitously falsified the real situation, will probably felicitate himself over his cunning. His triumph, however, will only be of a temporary nature. "*Mene, mene, tekel*

*upharsin*" was never more plainly written than the handwriting on the wall with reference to the so-called ethics, of which it seems that the loudest and most ardent champions are the earliest to violate either in letter or in spirit.

Dr. Love, the distinguished second vice-president of the American Medical Association, has definitely and intelligently set forth the proper attitude of the profession to ethics, in his address, published in the July number of the JOURNAL, and which we need not further dilate upon at present. Let all interested read Dr. Love's address.

---

THE Pan-American Medical Congress, which has excited such universal interest in medical circles for the past two years, is nearly at hand. It will be held at Washington, Tuesday, Wednesday, Thursday, and Friday, September 5, 6, 7, and 8, 1893. Let us again urge all progressive physicians to attend this remarkable meeting, and to take part in its deliberations.

Rarely has it been the province of the profession to consider in Medical Congress assembled more important issues than will be discussed at this meeting. International quarantine, marine sanitation, and medical pedagogics, together with all other scientific medical questions, will be broadly discussed.

We publish in this issue of the JOURNAL the program, in part, of the Section on Gynecology and Abdominal Surgery. A number of papers have been announced since this program was put in type, and it is safe to say that the work to be done by this Section will take high rank in the literature of the departments of medicine which is its province to consider.

---

THE Post-office building in Buffalo is a fair target for the Health Commissioner in his endeavor to improve the sanitary condition of this city. Let him visit the basement and there make note of the environment of the faithful officials who conduct the most important branch of the postal service. If foul air, superheated by gas, can ever breed disease, it would seem that here is a favorable opportunity.

The New York Board of Health has made an inspection of the post-office building in that city, where there are 259 persons employed in the newspaper department in the cellar of the building, of whom there are on an average of ten on the sick list daily.

While these buildings are under the control of the general government, it is yet within the province of the local boards of health to make recommendations looking towards the improvement of the present unsanitary conditions. Let such a report be made with reference to the Post-office building in Buffalo, and forward it to Postmaster Gentsch. A copy of such report would, no doubt, be forwarded by him to the Washington authorities, and this would, probably, hasten action with reference to the erection of a post-office building more than any other argument.

---

THE eleventh International Medical Congress, that was to have convened in Rome in the latter part of September, has been postponed until April of next year, on account of the prevalence of cholera in Italy. This, it seems to us, is a wise solution of the problem. No doubt there will be much disappointment, in view of the fact that many of the members of the Pan-American Medical Congress, which will meet in Washington, September 5th, had arranged for an excursion to Rome by the Werra, to sail from New York on September 9th; but this is of too small a moment as compared with the danger that might grow out of the assembling of the Congress during such an epidemic.

